# Isopropyl 4-amino­benzoate

**DOI:** 10.1107/S241431462200904X

**Published:** 2022-09-15

**Authors:** Prabhakar Priyanka, Bidarur K. Jayanna, Haruvegowda Kiran Kumar, Hemmige S. Yathirajan, Thayamma R. Divakara, Sabine Foro, Ray J. Butcher

**Affiliations:** a Department of Chemistry, B. N. M. Institute of Technology, Bengaluru 560 070, India; b Department of Studies in Chemistry, University of Mysore, Manasagangotri, Mysuru 570 006, India; c T. John Institute of Technology, Begaluru 560 083, India; d Institute of Materials Science, Darmstadt University of Technology, Petersenstrasse 23, D-64287 Darmstadt, Germany; e Department of Chemistry, Howard University, 525 College Street NW, Washington DC 20059, USA; University of Aberdeen, Scotland

**Keywords:** crystal structure, hydrogen bond, conformation

## Abstract

The title compound, C_10_H_13_NO_2_, crystallizes with two mol­ecules (*A* and *B*) in the asymmetric unit. For *A*, the dihedral angle between the plane of the phenyl ring and the *i*-propyl substituent is 65.4 (3)° while for ***B*** this angle is 67.8 (3)°. In the crystal, the mol­ecules are linked by N—H⋯O and N—H⋯N hydrogen bonds to generate double chains propagating in the [100] direction.

## Abstract 2

The title compound crystallizes with two mol­ecules in the asymmetric unit, which form double chains in the crystal linked by N—H⋯O and N—H⋯N hydrogen bonds.

## Graphical abstract

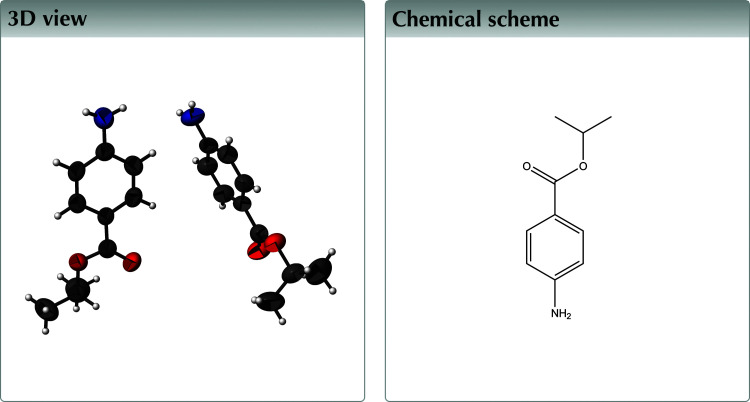

## Structure description

Isopropyl 4-amino­benzoate, C_10_H_13_NO_2_, serves as a model drug in correlation studies between HPLC retention parameters and percutaneous absorption (Fu & Liang, 1994 ▸). It functions as an inhibitor or an alternative acceptor substrate in the enzymatic acetyl­ation of *p*-nitro­aniline (Hanna *et al.*, 1990 ▸). The related compound risocaine (propyl 4-amino­benzoate) is a local anesthetic (Imai *et al.*, 2006 ▸), whereas benzocaine (ethyl 4-amino­benzoate) is utilized as a topical pain reliever (Fischer & Ganellin, 2006 ▸).

Some related crystal structures *viz*., the monoclinic form of ethyl 4-amino­benzoate (Lynch & McClenaghan, 2002 ▸), form (II) of benzocaine (Chan *et al.*, 2009 ▸; Chan & Welberry, 2010 ▸), 4-methyl­benzyl 4-amino­benzoate (Haider *et al.*, 2010 ▸), 2-(di­methyl­amino)­ethyl 4-amino­benzoate (Li *et al.*, 2019 ▸) and a new high-pressure benzocaine polymorph (Patyk-Kaźmierczak & Kaźmierczak, 2020 ▸) have been reported.

**Figure 1:**
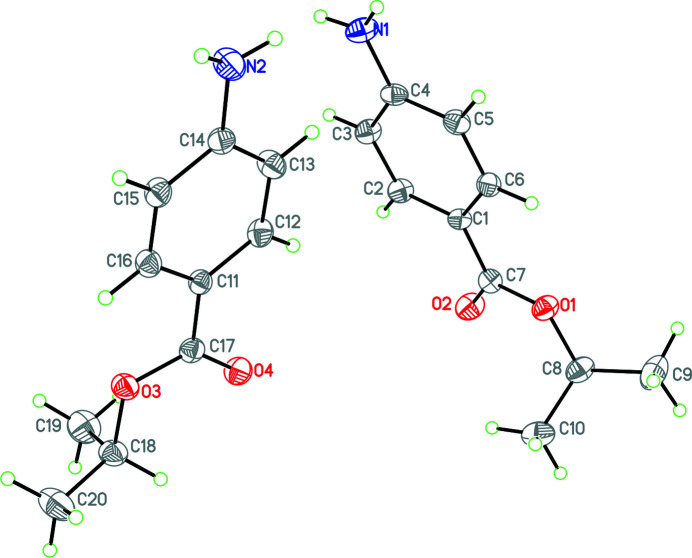
The mol­ecular structure of **I** with displacement ellipsoids drawn at the 30% probability level.

**Figure 2:**
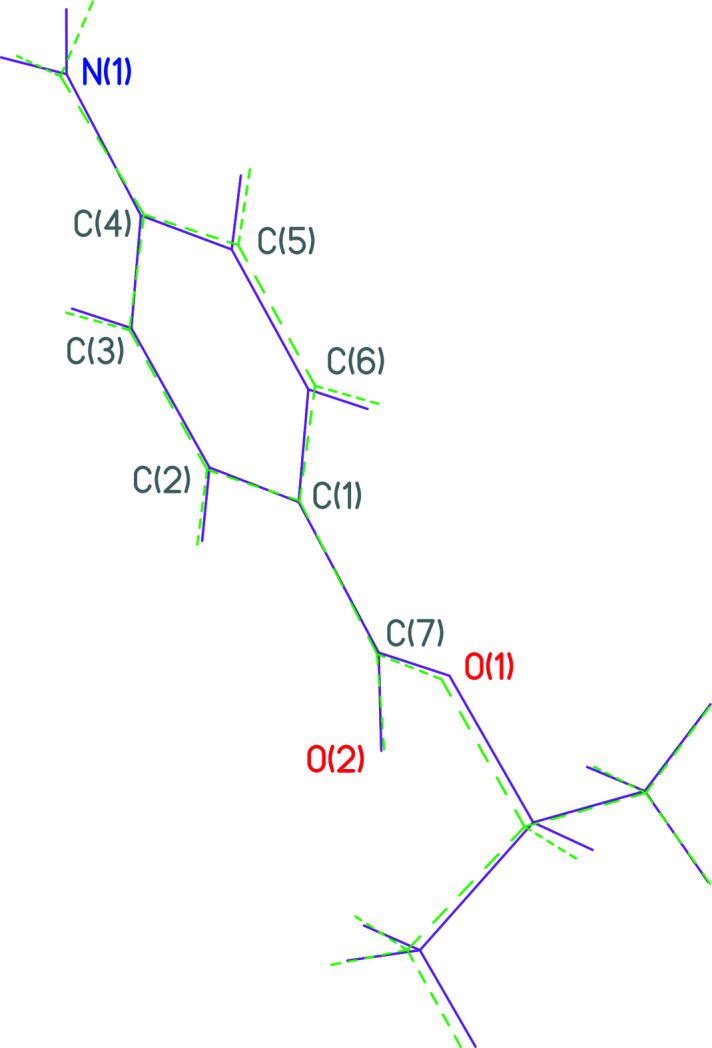
An overlap of mol­ecules *A* and *B* centered on the phenyl rings of both mol­ecules showing the differences in both conformers involving the conformations of both the NH_2_ and *i*-propyl substituents.

The present paper reports the synthesis and crystal structure of the title compound, (**I**). Compound **I** crystallizes with two mol­ecules in the asymmetric unit (Fig. 1 ▸). There are slight differences in the conformations of each mol­ecule: for *A*, the dihedral angle between the planes of the phenyl ring and its i-propyl substituent is 65.4 (3)° while for *B* this angle is 67.8 (3)°. For both mol­ecules, the H atoms of the amino substituents are not coplanar with their attached phenyl ring. This is indicated by the dihedral angles between this group and its phenyl ring [11.5 (3) and 24.2 (5)° for *A* and *B*, respectively] and the sum of the angles subtended at the N (358 and 352° for *A* and *B*, respectively), which shows that N2 is slightly more pyramidal than N1. These differences in the conformations of *A* and *B* are most clearly shown in an overlay of both mol­ecules centered on the phenyl ring of both (Fig. 2 ▸).

**Figure 3:**
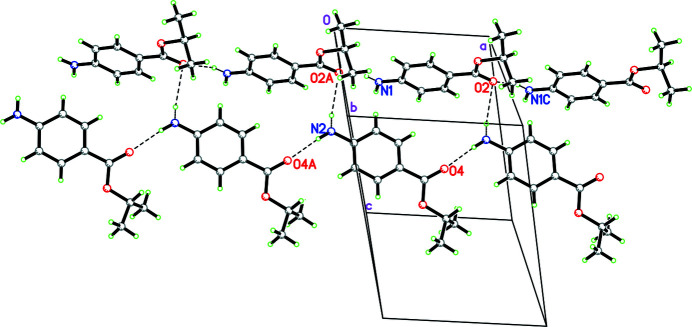
Partial packing diagram for **I** showing the formation of [100] double chains linked by N—H⋯O and N—H⋯N hydrogen bonds (shown as dashed lines).

In the extended structure of **I**, the mol­ecules are linked by N—H⋯O and N—H⋯N hydrogen bonds (Table 1 ▸) to generate double chains propagating in the [100] direction (Fig. 2 ▸). The chains consist of *A*⋯*A*⋯*A* and *B*⋯*B*⋯*B* mol­ecules linked by N1—H11*N*⋯O2 and N2—H21*N*⋯O4 hydrogen bonds, respectively, which both generate *C*(8) chains, with the N1—H12*N*⋯O2 and N2—H22*N*⋯O hydrogen bonds cross-linking the chains (Fig. 3 ▸).

## Synthesis and crystallization

**Figure 4:**
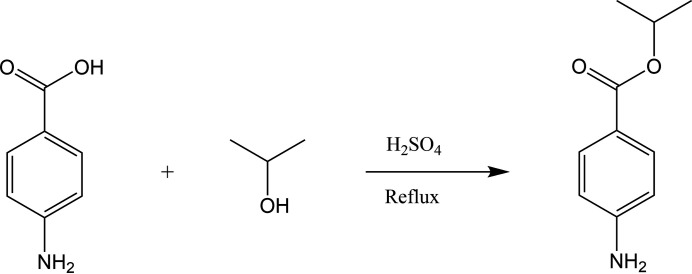
Reaction scheme.

4-Amino­benzoic acid (1.0 g), purchased from Sigma–Aldrich, was taken in a 100 ml round-bottomed flask. Then, 20 ml of 2-propanol and a catalytic amount of conc. H_2_SO_4_ was added and the reaction mixture was refluxed for 4 h. The reaction was confirmed to be complete using thin-layer chromatography and the mixture was then quenched with water, the precipitate formed was collected by filtration and dried. Pink needles suitable for single-crystal X-ray diffraction were grown by slow evaporation, at room temperature of a solution in ethyl acetate. Yield (79%), m. p. 355–357 K. The reaction scheme is shown in Fig. 4 ▸.

## Refinement

Crystal data, data collection and structure refinement details for **I** are summarized in Table 2 ▸.

## Supplementary Material

Crystal structure: contains datablock(s) I. DOI: 10.1107/S241431462200904X/hb4410sup1.cif

Structure factors: contains datablock(s) I. DOI: 10.1107/S241431462200904X/hb4410Isup2.hkl

Supporting information file. DOI: 10.1107/S241431462200904X/hb4410Isup3.cml

CCDC reference: 2206385

Additional supporting information:  crystallographic information; 3D view; checkCIF report

## Figures and Tables

**Table 1:** Hydrogen-bond geometry (Å, °)

*D*—H⋯*A*	*D*—H	H⋯*A*	*D*⋯*A*	*D*—H⋯*A*
N1—H11*N*⋯O2^i^	0.87 (2)	2.23 (2)	3.060 (4)	158 (3)
N1—H12*N*⋯N2^ii^	0.88 (2)	2.39 (2)	3.269 (5)	176 (4)
N2—H21*N*⋯O4^i^	0.87 (2)	2.07 (2)	2.930 (4)	168 (4)
N2—H22*N*⋯O2^i^	0.87 (2)	2.36 (2)	3.224 (5)	172 (4)

Symmetry codes: (i) 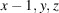 ; (ii) 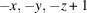 .

**Table 2:** Experimental details

Crystal data	
Chemical formula	C_10_H_13_NO_2_
*M*_r_	179.21
Crystal system, space group	Triclinic, *P* 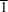
Temperature (K)	296
*a*, *b*, *c* (Å)	8.405 (1), 11.029 (2), 11.520 (3)
α, β, γ (°)	89.10 (2), 77.06 (2), 87.17 (2)
*V* (Å^3^)	1039.5 (4)
*Z*	4
Radiation type	Mo *K*α
μ (mm^−1^)	0.08
Crystal size (mm)	0.48 × 0.10 × 0.06

Data collection	
Diffractometer	Oxford Diffraction Xcalibur CCD
Absorption correction	Multi-scan (*CrysAlis RED*; Oxford Diffraction, 2009 ▸)
*T*_min_, *T*_max_	0.461, 1.000
No. of measured, independent and observed [*I* > 2σ(*I*)] reflections	6595, 3730, 1275
*R*_int_	0.062
(sin θ/λ)_max_ (Å^−1^)	0.600

Refinement	
*R*[*F* ^2^ > 2σ(*F* ^2^)], *wR*(*F* ^2^), *S*	0.083, 0.135, 0.98
No. of reflections	3730
No. of parameters	251
No. of restraints	4
H-atom treatment	H atoms treated by a mixture of independent and constrained refinement
Δρ_max_, Δρ_min_ (e Å^−3^)	0.11, −0.14

Computer programs: *CrysAlis CCD* (Oxford Diffraction, 2009 ▸), *CrysAlis RED* (Oxford Diffraction, 2009 ▸), *SHELXT* (Sheldrick, 2015*a* ▸), *SHELXL2014/6* (Sheldrick, 2015*b* ▸) and *SHELXTL* (Sheldrick, 2008 ▸).

## full crystallographic data

### Crystal data

**Table:**

C_10_H_13_NO_2_	*Z* = 4
*M**_r_* = 179.21	*F*(000) = 384
Triclinic, *P*1	*D*_x_ = 1.145 Mg m^−^^3^
*a* = 8.405 (1) Å	Mo *K*α radiation, λ = 0.71073 Å
*b* = 11.029 (2) Å	Cell parameters from 837 reflections
*c* = 11.520 (3) Å	θ = 2.6–28.0°
α = 89.10 (2)°	µ = 0.08 mm^−^^1^
β = 77.06 (2)°	*T* = 296 K
γ = 87.17 (2)°	Needle, pink
*V* = 1039.5 (4) Å^3^	0.48 × 0.10 × 0.06 mm

### Data collection

**Table:**

Oxford Diffraction Xcalibur CCD diffractometer	1275 reflections with *I* > 2σ(*I*)
Radiation source: Enhance (Mo) X-ray Source	*R*_int_ = 0.062
ω scans	θ_max_ = 25.3°, θ_min_ = 2.6°
Absorption correction: multi-scan (CrysalisRed; Oxford Diffraction, 2009)	*h* = −10→9
*T*_min_ = 0.461, *T*_max_ = 1.000	*k* = −7→13
6595 measured reflections	*l* = −13→13
3730 independent reflections	

### Refinement

**Table:**

Refinement on *F*^2^	Primary atom site location: dual
Least-squares matrix: full	Secondary atom site location: difference Fourier map
*R*[*F*^2^ > 2σ(*F*^2^)] = 0.083	Hydrogen site location: mixed
*wR*(*F*^2^) = 0.135	H atoms treated by a mixture of independent and constrained refinement
*S* = 0.98	*w* = 1/[σ^2^(*F*_o_^2^) + (0.0372*P*)^2^] where *P* = (*F*_o_^2^ + 2*F*_c_^2^)/3
3730 reflections	(Δ/σ)_max_ < 0.001
251 parameters	Δρ_max_ = 0.11 e Å^−^^3^
4 restraints	Δρ_min_ = −0.14 e Å^−^^3^

### Special details

**Table:**

Geometry. All esds (except the esd in the dihedral angle between two l.s. planes) are estimated using the full covariance matrix. The cell esds are taken into account individually in the estimation of esds in distances, angles and torsion angles; correlations between esds in cell parameters are only used when they are defined by crystal symmetry. An approximate (isotropic) treatment of cell esds is used for estimating esds involving l.s. planes.
Refinement. All hydrogen atoms were placed geometrically and refined as riding atoms with their *U*_iso_ values 1.2 times (1.5 times for CH_3_) that of their attached atoms.

### Fractional atomic coordinates and isotropic or equivalent isotropic displacement parameters (Å^2^)

**Table:**

	*x*	*y*	*z*	*U*_iso_*/*U*_eq_	
O1	0.8880 (3)	0.1710 (2)	0.0611 (2)	0.0724 (8)	
O2	0.9815 (3)	0.0696 (2)	0.2037 (2)	0.0859 (10)	
N1	0.2362 (5)	−0.0792 (3)	0.3053 (3)	0.0825 (11)	
H11N	0.160 (4)	−0.056 (3)	0.268 (3)	0.099*	
H12N	0.207 (4)	−0.116 (3)	0.374 (2)	0.099*	
C1	0.7041 (4)	0.0527 (3)	0.1917 (3)	0.0520 (10)	
C2	0.6632 (5)	−0.0177 (3)	0.2936 (4)	0.0677 (12)	
H2	0.742114	−0.036394	0.337017	0.081*	
C3	0.5097 (5)	−0.0607 (3)	0.3327 (3)	0.0679 (12)	
H3	0.486052	−0.107475	0.401760	0.082*	
C4	0.3891 (5)	−0.0345 (3)	0.2689 (4)	0.0604 (11)	
C5	0.4306 (5)	0.0348 (3)	0.1661 (4)	0.0666 (11)	
H5	0.352592	0.052365	0.121639	0.080*	
C6	0.5839 (5)	0.0780 (3)	0.1283 (3)	0.0609 (11)	
H6	0.607661	0.124870	0.059323	0.073*	
C7	0.8696 (5)	0.0957 (3)	0.1551 (4)	0.0627 (11)	
C8	1.0471 (5)	0.2228 (4)	0.0148 (4)	0.0802 (13)	
H8	1.133590	0.159715	0.013890	0.096*	
C9	1.0485 (5)	0.2620 (4)	−0.1104 (4)	0.1137 (16)	
H9A	1.153638	0.291440	−0.146885	0.171*	
H9B	0.965887	0.325395	−0.109877	0.171*	
H9C	1.026744	0.194107	−0.154764	0.171*	
C10	1.0704 (5)	0.3246 (4)	0.0924 (4)	0.1263 (18)	
H10A	1.176583	0.355914	0.063178	0.190*	
H10B	1.061486	0.295363	0.172414	0.190*	
H10C	0.988098	0.387900	0.091451	0.190*	
O3	0.3779 (3)	0.4487 (2)	0.6988 (3)	0.0712 (8)	
O4	0.5570 (3)	0.3581 (2)	0.5474 (2)	0.0782 (9)	
N2	−0.1326 (5)	0.2278 (4)	0.4435 (3)	0.0884 (12)	
H21N	−0.223 (3)	0.273 (3)	0.465 (3)	0.106*	
H22N	−0.113 (5)	0.185 (3)	0.379 (2)	0.106*	
C11	0.2710 (5)	0.3471 (3)	0.5583 (4)	0.0539 (10)	
C12	0.2909 (5)	0.2638 (4)	0.4678 (4)	0.0657 (11)	
H12	0.395305	0.233201	0.432496	0.079*	
C13	0.1594 (5)	0.2250 (3)	0.4288 (4)	0.0727 (13)	
H13	0.175830	0.167394	0.368659	0.087*	
C14	0.0023 (6)	0.2703 (4)	0.4777 (4)	0.0624 (11)	
C15	−0.0176 (5)	0.3553 (4)	0.5675 (3)	0.0658 (12)	
H15	−0.121799	0.387177	0.601576	0.079*	
C16	0.1145 (5)	0.3934 (3)	0.6073 (3)	0.0637 (11)	
H16	0.098348	0.450695	0.667688	0.076*	
C17	0.4164 (6)	0.3833 (3)	0.5982 (4)	0.0607 (12)	
C18	0.5132 (5)	0.4917 (4)	0.7453 (4)	0.0777 (13)	
H18	0.604797	0.510435	0.679401	0.093*	
C19	0.5659 (5)	0.3927 (4)	0.8238 (4)	0.1091 (16)	
H19A	0.651956	0.420771	0.857562	0.164*	
H19B	0.604601	0.321671	0.776874	0.164*	
H19C	0.474575	0.373254	0.886569	0.164*	
C20	0.4458 (5)	0.6058 (4)	0.8133 (4)	0.1175 (17)	
H20A	0.531415	0.642247	0.841449	0.176*	
H20B	0.359770	0.585874	0.879823	0.176*	
H20C	0.403548	0.661803	0.761739	0.176*	

### Atomic displacement parameters (Å^2^)

**Table:**

	*U*^11^	*U*^22^	*U*^33^	*U*^12^	*U*^13^	*U*^23^
O1	0.062 (2)	0.0778 (19)	0.079 (2)	−0.0114 (15)	−0.0180 (16)	0.0140 (16)
O2	0.063 (2)	0.102 (2)	0.100 (2)	−0.0073 (16)	−0.0353 (18)	0.0239 (17)
N1	0.062 (3)	0.079 (3)	0.110 (3)	−0.014 (2)	−0.025 (3)	0.015 (2)
C1	0.050 (3)	0.047 (2)	0.063 (3)	−0.001 (2)	−0.021 (2)	0.002 (2)
C2	0.065 (3)	0.070 (3)	0.074 (3)	−0.004 (2)	−0.029 (3)	0.012 (3)
C3	0.068 (3)	0.063 (3)	0.075 (3)	−0.003 (2)	−0.023 (3)	0.014 (2)
C4	0.055 (3)	0.046 (3)	0.082 (3)	−0.001 (2)	−0.018 (3)	−0.007 (2)
C5	0.065 (3)	0.068 (3)	0.072 (3)	−0.001 (2)	−0.027 (3)	−0.001 (2)
C6	0.066 (3)	0.059 (3)	0.060 (3)	−0.004 (2)	−0.019 (3)	0.004 (2)
C7	0.064 (3)	0.060 (3)	0.065 (3)	0.003 (2)	−0.017 (3)	−0.001 (2)
C8	0.062 (3)	0.083 (3)	0.095 (4)	−0.011 (3)	−0.016 (3)	0.019 (3)
C9	0.105 (4)	0.140 (4)	0.091 (4)	−0.021 (3)	−0.009 (3)	0.040 (3)
C10	0.130 (5)	0.132 (4)	0.125 (4)	−0.066 (3)	−0.033 (4)	0.004 (4)
O3	0.0559 (19)	0.081 (2)	0.078 (2)	−0.0044 (15)	−0.0158 (17)	−0.0163 (17)
O4	0.0491 (18)	0.088 (2)	0.092 (2)	0.0018 (16)	−0.0030 (17)	−0.0148 (16)
N2	0.063 (3)	0.112 (4)	0.091 (3)	0.005 (2)	−0.019 (3)	−0.032 (2)
C11	0.050 (3)	0.055 (3)	0.055 (3)	0.001 (2)	−0.008 (2)	−0.001 (2)
C12	0.050 (3)	0.075 (3)	0.068 (3)	0.007 (2)	−0.007 (2)	−0.003 (3)
C13	0.061 (3)	0.080 (3)	0.079 (3)	0.008 (3)	−0.020 (3)	−0.020 (2)
C14	0.055 (3)	0.068 (3)	0.065 (3)	−0.004 (3)	−0.014 (3)	−0.001 (2)
C15	0.046 (3)	0.078 (3)	0.069 (3)	0.007 (2)	−0.005 (2)	−0.009 (3)
C16	0.064 (3)	0.060 (3)	0.064 (3)	0.003 (3)	−0.009 (3)	−0.006 (2)
C17	0.066 (3)	0.048 (3)	0.065 (3)	−0.001 (3)	−0.009 (3)	0.006 (2)
C18	0.062 (3)	0.084 (3)	0.089 (3)	−0.010 (3)	−0.020 (3)	−0.013 (3)
C19	0.104 (4)	0.125 (4)	0.109 (4)	0.001 (3)	−0.048 (3)	−0.003 (3)
C20	0.107 (4)	0.108 (4)	0.142 (5)	0.005 (3)	−0.035 (3)	−0.052 (4)

### Geometric parameters (Å, º)

**Table:**

O1—C7	1.340 (4)	O3—C17	1.343 (4)
O1—C8	1.465 (4)	O3—C18	1.462 (4)
O2—C7	1.218 (4)	O4—C17	1.216 (4)
N1—C4	1.373 (5)	N2—C14	1.386 (5)
N1—H11N	0.870 (18)	N2—H21N	0.873 (18)
N1—H12N	0.876 (18)	N2—H22N	0.866 (18)
C1—C2	1.384 (4)	C11—C12	1.377 (4)
C1—C6	1.389 (4)	C11—C16	1.386 (4)
C1—C7	1.461 (5)	C11—C17	1.472 (5)
C2—C3	1.373 (4)	C12—C13	1.372 (4)
C2—H2	0.9300	C12—H12	0.9300
C3—C4	1.396 (4)	C13—C14	1.386 (5)
C3—H3	0.9300	C13—H13	0.9300
C4—C5	1.386 (5)	C14—C15	1.383 (4)
C5—C6	1.371 (4)	C15—C16	1.378 (4)
C5—H5	0.9300	C15—H15	0.9300
C6—H6	0.9300	C16—H16	0.9300
C8—C10	1.493 (5)	C18—C20	1.510 (4)
C8—C9	1.497 (5)	C18—C19	1.518 (4)
C8—H8	0.9800	C18—H18	0.9800
C9—H9A	0.9600	C19—H19A	0.9600
C9—H9B	0.9600	C19—H19B	0.9600
C9—H9C	0.9600	C19—H19C	0.9600
C10—H10A	0.9600	C20—H20A	0.9600
C10—H10B	0.9600	C20—H20B	0.9600
C10—H10C	0.9600	C20—H20C	0.9600
			
C7—O1—C8	119.2 (3)	C17—O3—C18	117.3 (3)
C4—N1—H11N	119 (3)	C14—N2—H21N	115 (3)
C4—N1—H12N	121 (3)	C14—N2—H22N	116 (3)
H11N—N1—H12N	118 (4)	H21N—N2—H22N	121 (4)
C2—C1—C6	117.5 (4)	C12—C11—C16	118.2 (4)
C2—C1—C7	119.4 (3)	C12—C11—C17	118.8 (4)
C6—C1—C7	123.1 (4)	C16—C11—C17	123.1 (4)
C3—C2—C1	122.1 (3)	C13—C12—C11	121.2 (4)
C3—C2—H2	118.9	C13—C12—H12	119.4
C1—C2—H2	118.9	C11—C12—H12	119.4
C2—C3—C4	120.1 (4)	C12—C13—C14	121.0 (4)
C2—C3—H3	119.9	C12—C13—H13	119.5
C4—C3—H3	119.9	C14—C13—H13	119.5
N1—C4—C5	121.4 (4)	C15—C14—N2	120.3 (4)
N1—C4—C3	120.9 (4)	C15—C14—C13	117.9 (4)
C5—C4—C3	117.8 (4)	N2—C14—C13	121.7 (4)
C6—C5—C4	121.6 (4)	C16—C15—C14	121.1 (4)
C6—C5—H5	119.2	C16—C15—H15	119.5
C4—C5—H5	119.2	C14—C15—H15	119.5
C5—C6—C1	120.9 (4)	C15—C16—C11	120.7 (4)
C5—C6—H6	119.6	C15—C16—H16	119.7
C1—C6—H6	119.6	C11—C16—H16	119.7
O2—C7—O1	121.8 (4)	O4—C17—O3	122.4 (4)
O2—C7—C1	125.3 (4)	O4—C17—C11	125.0 (4)
O1—C7—C1	112.9 (4)	O3—C17—C11	112.6 (4)
O1—C8—C10	110.1 (4)	O3—C18—C20	105.2 (3)
O1—C8—C9	106.2 (3)	O3—C18—C19	108.4 (3)
C10—C8—C9	113.1 (4)	C20—C18—C19	112.8 (4)
O1—C8—H8	109.1	O3—C18—H18	110.1
C10—C8—H8	109.1	C20—C18—H18	110.1
C9—C8—H8	109.1	C19—C18—H18	110.1
C8—C9—H9A	109.5	C18—C19—H19A	109.5
C8—C9—H9B	109.5	C18—C19—H19B	109.5
H9A—C9—H9B	109.5	H19A—C19—H19B	109.5
C8—C9—H9C	109.5	C18—C19—H19C	109.5
H9A—C9—H9C	109.5	H19A—C19—H19C	109.5
H9B—C9—H9C	109.5	H19B—C19—H19C	109.5
C8—C10—H10A	109.5	C18—C20—H20A	109.5
C8—C10—H10B	109.5	C18—C20—H20B	109.5
H10A—C10—H10B	109.5	H20A—C20—H20B	109.5
C8—C10—H10C	109.5	C18—C20—H20C	109.5
H10A—C10—H10C	109.5	H20A—C20—H20C	109.5
H10B—C10—H10C	109.5	H20B—C20—H20C	109.5
			
C6—C1—C2—C3	−0.6 (5)	C16—C11—C12—C13	−1.5 (5)
C7—C1—C2—C3	179.9 (3)	C17—C11—C12—C13	178.7 (3)
C1—C2—C3—C4	0.3 (6)	C11—C12—C13—C14	1.3 (5)
C2—C3—C4—N1	178.5 (4)	C12—C13—C14—C15	−0.4 (5)
C2—C3—C4—C5	0.4 (5)	C12—C13—C14—N2	−176.8 (4)
N1—C4—C5—C6	−178.9 (4)	N2—C14—C15—C16	176.3 (3)
C3—C4—C5—C6	−0.9 (5)	C13—C14—C15—C16	−0.2 (5)
C4—C5—C6—C1	0.6 (6)	C14—C15—C16—C11	−0.1 (5)
C2—C1—C6—C5	0.1 (5)	C12—C11—C16—C15	1.0 (5)
C7—C1—C6—C5	179.6 (3)	C17—C11—C16—C15	−179.3 (3)
C8—O1—C7—O2	0.4 (5)	C18—O3—C17—O4	1.3 (5)
C8—O1—C7—C1	179.3 (3)	C18—O3—C17—C11	−178.8 (3)
C2—C1—C7—O2	4.2 (6)	C12—C11—C17—O4	10.3 (5)
C6—C1—C7—O2	−175.2 (4)	C16—C11—C17—O4	−169.4 (4)
C2—C1—C7—O1	−174.6 (3)	C12—C11—C17—O3	−169.6 (3)
C6—C1—C7—O1	5.9 (5)	C16—C11—C17—O3	10.6 (5)
C7—O1—C8—C10	−77.8 (4)	C17—O3—C18—C20	152.5 (3)
C7—O1—C8—C9	159.5 (3)	C17—O3—C18—C19	−86.6 (4)

### Hydrogen-bond geometry (Å, º)

**Table:**

*D*—H···*A*	*D*—H	H···*A*	*D*···*A*	*D*—H···*A*
N1—H11*N*···O2^i^	0.87 (2)	2.23 (2)	3.060 (4)	158 (3)
N1—H12*N*···N2^ii^	0.88 (2)	2.39 (2)	3.269 (5)	176 (4)
N2—H21*N*···O4^i^	0.87 (2)	2.07 (2)	2.930 (4)	168 (4)
N2—H22*N*···O2^i^	0.87 (2)	2.36 (2)	3.224 (5)	172 (4)

Symmetry codes: (i) *x*−1, *y*, *z*; (ii) −*x*, −*y*, −*z*+1.
